# The Role of Serine-Threonine Kinase Receptor-Associated Protein (STRAP) Signaling in Cancer

**DOI:** 10.3390/cells14120854

**Published:** 2025-06-06

**Authors:** Sourajeet Karfa, Shashank Saurav, Bryan Feng, Song Li, Brian K. Law, Pran K. Datta

**Affiliations:** 1 Division of Hematology and Oncology, Department of Medicine, UAB Comprehensive Cancer Center, University of Alabama at Birmingham, Birmingham, AL 35294, USA; skarfa@uab.edu (S.K.); ssaurav@uabmc.edu (S.S.);; 2 Center for Pharmacogenetics, Department of Pharmaceutical Sciences, University of Pittsburgh School of Pharmacy, Pittsburgh, PA 15261, USA; sol4@pitt.edu; 3 Department of Pharmacology and Therapeutics, University of Florida, Gainesville, FL 32610, USA; bklaw@ufl.edu; 4 Birmingham Veterans Affairs Health Care System, Birmingham, AL 35233, USA

**Keywords:** cancer, STRAP, TGF-ß, signaling, cell proliferation, apoptosis, oncogenesis

## Abstract

STRAP (serine-threonine kinase receptor-associated protein), a WD domain-containing 38.5 kDa protein, was first identified in TGF-ß signaling and participates in scaffold formation in numerous cellular multiprotein complexes. It is involved in the regulation of several oncogenic biological processes, including cell proliferation, apoptosis, migration/invasion, tumor initiation and progression, and metastasis. STRAP upregulation in epithelial tumors regulates several signaling pathways, such as TGF-ß, MEK/ERK, Wnt/β-Catenin, Notch, PI3K, NF-κB, and ASK-1 in human cancers, including colon, breast, lung, osteosarcoma, and neuroblastoma. The upregulation of STRAP expression is correlated with worse survival in colorectal cancer following post-adjuvant therapy. Strap knockout sensitizes colon tumors to chemotherapy, delays APC-induced tumor progression, and reduces cancer cell stemness. The loss of *Strap* disrupts lineage differentiation, delays neural tube closure, and alters exon skipping, resulting in early embryonic lethality in mice. Collectively, the purpose of this review is to update and describe the diversity of targets functionally interacting with STRAP and to rationalize the involvement of STRAP in a variety of signaling pathways and biological processes. Therefore, these in vitro and in vivo studies provide a proof of concept that lowering STRAP expression in solid tumors decreases tumorigenicity and metastasis, and targeting STRAP provides strong translational potential to develop pre-therapeutic leads.

## 1. Introduction

The proliferation of cancer cells is the fundamental abnormality leading to the development of cancer. Genetic mutations and epigenetic changes often lead to the deregulation of cell signaling and aberrant molecular mechanisms, resulting in tumor initiation, progression, metastasis, and chemoresistance. The serine-threonine kinase receptor-associated protein (STRAP) was first identified as a TGF-ß type I receptor (TßR-I, a serine-threonine kinase receptor)-associated protein. It is a ubiquitous WD40 domain-containing, predominantly cytoplasmic protein. It was initially cloned and identified as an inhibitor of TGF-β-mediated Smad2/3 phosphorylation and transcriptional regulation [1,2]. In normal cellular physiology, STRAP regulates cell growth, maintenance, and nuclear export through SMN complex-mediated alternate RNA splicing [3,4]. STRAP is upregulated in 70% of colon, 78% of lung [5], and 60% of breast cancer [6] and is primarily expressed in transformed epithelial cells, suggesting its potential role as an oncogenic protein. In colorectal cancer patients, STRAP expression has been found to be highest in Stage I, and its levels are maintained in Stage II/III/IV, where STRAP may contribute to its oncogenic and chemoresistance functions.

In general, WD40 domain-containing proteins appear to be involved in a variety of cellular processes, such as signal transduction, transcriptional regulation, RNA processing, vesicular trafficking, cell cycle progression, programmed cell death, and protein stability. Research has demonstrated that STRAP plays a role in the amount of cell growth and cancer progression pathways [5,7,8]. The WD40 domain proteins form a β-propeller architecture, and these domains are known as beta-transducing domains with around 40 amino acids ending with Tryptophan (W) and Aspartic acid (D) [9]. WD40 domain-containing proteins are evolutionarily conserved and present in prokaryotes and lower eukaryotes [10,11]. Most of the WD40 domain-containing proteins are scaffolds for protein–protein or protein–peptide interaction and regulate downstream molecular pathways that control signaling, ubiquitination, histone methylation, and chromatin remodeling [9]. STRAP acts as a scaffold by interacting with various key molecules involved in cell-fate-deciding pathways such as TGF-β, Wnt/ß-Catenin, Notch, etc. STRAP upregulation has been shown to attenuate TGF-β-mediated growth inhibition in lung and colon carcinoma and increase tumorigenesis in xenograft models [5,12]. The upregulation of STRAP correlates with ICN3 (intracellular fragment of Notch3) in 59% of non-small-cell lung cancers and increases its stabilization [13]. As an upstream regulator of MEK/ERK and Wnt/β-Catenin signaling in intestinal cancers, STRAP plays an important role in promoting APC-induced tumor development and progression [14]. In cancer cells, STRAP has been found to be involved in several signal transduction pathways, including TGF-β, NOTCH, MEK/ERK, and Wnt/β-Catenin, that may contribute to signal transduction, transcriptional regulation, programmed cell death, EMT, invasion, RNA processing, chromatin assembly, cell cycle progression, and vesicular trafficking [5,7,8,15]. Therefore, the upregulation of STRAP induces cell proliferation and survival by impeding cell cycle arrest and apoptosis in normal and cancer cells, suggesting its prooncogenic activities.

Although increased levels of STRAP have been correlated with cancer progression, its homozygous deletion resulted in mouse embryonic lethality and morphological defects, causing death at days E9.5 and E10.5 due to defects in angiogenesis, cardiogenesis, somitogenesis, and neural tube closure, suggesting its indispensable functions in embryonic development [16,17,18]. With the due course of scientific advancement, STRAP has been involved in various pathways to regulate cell growth and maintenance. In the current review, we have discussed the oncogenic roles of cell growth, differentiation, EMT, cancer initiation/progression, and chemoresistance.

## 2. STRAP: Structure, Domain, and Functional Interactions

STRAP is a 38.5 kDa protein (350 amino acids) that is a member of the WD40 protein family. It comprises seven WD40 domains containing approximately 40 amino acids and forms a circularized β-propeller structure [1,19]. The Strap gene was initially cloned from a mouse embryonic cDNA library using yeast two-hybrid screening with TßR-I as a bait. Later, it was shown that both type I and type II serine-threonine kinase receptors of TGF-ß bind STRAP [1,2]. The Strap gene is widely distributed in eukaryotes from humans to lower eukaryotes, such as yeast. It has shown 55% homology in base pairs and 19% similarity in amino acids to another WD40 protein, TRIP1 [19,20]. In humans, the STRAP gene is located on chromosome 12p12.3 near marker D12S1593. Later, the human version of the Strap gene (HMAWD) was identified, which was 97% identical to STRAP in amino acid sequences [6,21]. It is ubiquitously expressed in all mouse tissues, with lower abundance in the spleen and highest levels in the liver and testes. In humans, STRAP expression is ubiquitous and forms a 2 kb transcript. In both humans and mice, the STRAP genes consist of 10 exons. STRAP is mostly localized in the cytoplasm and is present at low levels in the nucleus. It forms homo-oligomers, probably through WD40 repeats, which are important for multiprotein complex assembly [2,5].

STRAP not only associates with TβR-I and TβR-II, but also interacts with Smad2, Smad3, Smad6, Smad7, 3-phosphoinositide-dependent protein kinase 1, nuclear export factor (NXF) proteins, Ewing’s sarcoma protein (EWS), hMAWD-binding protein (MAWBP), Gemin6, Gemin7, unr, microtubule-associated protein 1B (MAP1B), and three small nuclear ribonucleoproteins: SmB, SmD2, and SmD3 [15]. Moreover, an ELM motif search also indicates potential binding to other proteins, including C-terminal-binding protein (CtBP), protein phosphatase 1, retinoblastoma protein (pRb), TNF receptor-associated factor 2 (TRAF-2), glycosaminoglycans, and proteins with class II PDZ domain, SH2 domain, and class IV WW domain [15].

## 3. Regulation of Oncogenic Signaling by STRAP: Mechanistic Insights

### 3.1. STRAP Inhibits Smad-Dependent TGF-ß Signaling

TGF-β signaling regulates a wide range of cellular processes like cell proliferation, differentiation, apoptosis, plasticity, and migration, and its disruption may result in various kinds of diseases, such as cancer and tissue fibrosis. In mammalian cells, upon binding of an active TGF-β ligand to TβR-II, TβR-I is recruited and phosphorylated by TβR-II in the cytoplasmic domain. The activated heterotetrameric TβR-I/TβR-II complex triggers the canonical Smad-dependent and noncanonical Smad-independent signaling pathways [22,23,24]. In Smad-dependent signaling, the activated TβR-I/TβR-II complex phosphorylates and activates receptor-associated SMADs (Smad2 and Smad3) [24,25,26,27]. The activated Smad2/3 complex binds Smad4 and is translocated to the nucleus by importin-B, where it binds with other transcription factors to initiate or suppress transcription of TGF-β target genes [19,23,24]. TGF-β/Smad signaling has contextual differences, but the Smad-dependent pathway is recognized as a potent suppressor of tumor formation, as the activation of Smads inhibits cell cycle progression and induces apoptosis [24].

**Figure 1:**
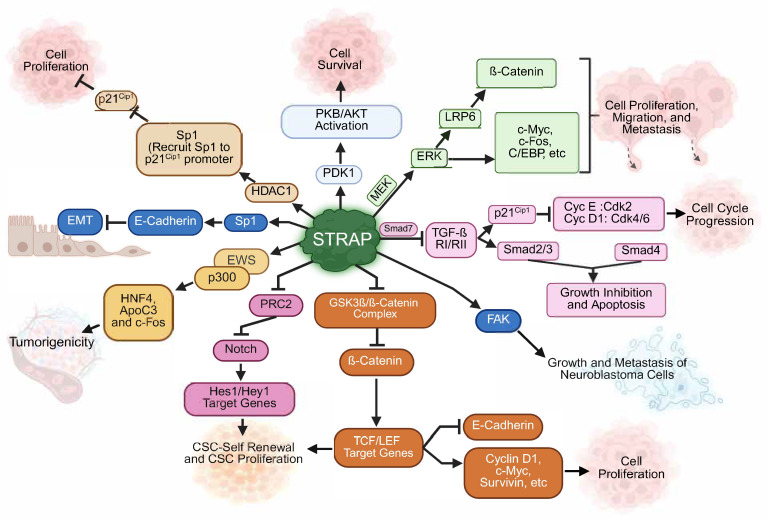
**STRAP-mediated key oncogenic signaling pathways in tumor progression, EMT, metastasis, and chemoresistance.** STRAP regulates multiple signaling pathways involved in cell proliferation, survival, epithelial–mesenchymal transition (EMT), cancer stem cell (CSC) renewal, and metastasis. STRAP recruits Sp1 and HDAC1 to the p21^Cip1^ promoter, decreasing p21^Cip1^ expression and modulating Cyclin E-CDK2 and Cyclin D1-CDK4/6 complexes to promote G1/S transition. As a result of activating the PI3K/AKT pathway through PDK1, STRAP enhances the survival of tumor cells by protecting them against apoptotic signals. STRAP stabilizes β-Catenin through its association with GSK3β. Stabilized β-Catenin translocates to the nucleus and activates oncogenic target genes, such as Cyclin D1, c-Myc, and Survivin, that are involved in driving tumor proliferation, migration, and metastasis. Through another mechanism, STRAP promotes the phosphorylation of ERK1/2 by forming a complex with MEK1/2, which in turn phosphorylates LRP6 and activates Wnt/ß-Catenin signaling. STRAP inhibits TGF-β signaling by recruiting Smad7 to the TGF-ß receptor complex (TGF-ß RI/RII) and influences EMT through E-cadherin regulation, thereby contributing to cancer progression and metastasis. Further, STRAP interacts with the Notch pathway to regulate Hes1/Hey1 target genes, thus influencing CSC renewal and proliferation. Together, STRAP orchestrates a complex network of oncogenic signaling pathways, emphasizing its critical role in the progression of cancer and its potential as a therapeutic target.

STRAP interacts with Smad7 and recruits it from the cytosol to the receptor complex, resulting in the formation of a multimeric complex. The formation of this complex inhibits the receptor-mediated phosphorylation of Smad2 and Smad3 [2,25] and nuclear translocation of the Smad2/3/4 complex. As a result, the TGF-ß target proteins PAI-1, p^15^, p^16^, and p^21^ are downregulated [23,24]. PAI-1, a natural inhibitor of tumor-cell-associated urokinase-type plasminogen activator (uPA), inhibits plasminogen conversion to plasmin and prevents angiogenesis and extracellular matrix degradation [24,28]. p^15^, p^16^, and p^21^ are cyclin-dependent kinase inhibitors (CDKI) that inhibit cyclin D1-CDK4/6 and/or cyclin E-CDK2 activity, blocking pRb phosphorylation and inducing cell cycle arrest at the G1-S transition [29]. In normal cellular physiology, inhibitory Smad6 and Smad7 (I-Smads) bind to TβR-I and inhibit R-Smads and their downstream transcriptional regulation [30,31]. STRAP has been shown to interact with TβR-I and/or TβR-II alone or along with Smad7, but not with Smad6, and inhibit Smad-mediated transcriptional regulation [24]. Smad6 inhibits bone morphogenic protein (BMP) signaling, but STRAP-Smad6 interaction rescues Smad6-mediated inhibitory effects [19]. Altogether, STRAP inhibits TGFβ-mediated Smad signaling through TβR-I/TβR-II and/or Smad6/7 interactions and contributes towards the growth-promoting activities of cancer cells (Figure 1).

### 3.2. STRAP Promotes Cell Growth by Activating PDK1 Signaling

3-phosphoinositide-dependent kinase-1 (PDK-1) is a serine-threonine kinase with an affinity for the lipid products of PI_3_K, PIP_2_, and PIP_3_. PDK1 phosphorylates a wide array of signal transduction proteins, including protein kinase C (PKC), protein kinase A (PKA), protein kinase G (PKG), S6 ribosomal kinase (S6K), p21 (Cdc42/Rac)-activated kinase 1 (PAK1), serum/glucocorticoid-regulated kinase (SGK), and Akt [32,33,34,35,36,37]. PDK-1/Akt signaling activation induces cellular proliferation, survival, and migration in pancreatic cells. PDK-1 inhibition has also been shown to prevent tamoxifen resistance in breast cancer [24,38,39,40,41]. STRAP interacts with PDK-1 and positively regulates its activity, resulting in the inhibition of TNFα- and/or TGFβ-mediated cell apoptosis. Since PDK-1 increases TβR-I-Smad7 interaction and inhibits the TGFβ-mediated signaling pathway, the co-overexpression/interaction of STRAP and PDK-1 negatively regulates TGFβ-mediated cell growth inhibition [42]. STRAP phosphorylation at Thr^175^ and Ser^179^ by ASK-1 leads to the inhibition of ASK-1-mediated cell death [43]. Therefore, under normal physiological conditions, STRAP promotes cell proliferation and survival and inhibits apoptosis (Figure 1).

### 3.3. STRAP Promotes EMT by Regulating E-Cadherin Expression and Attenuates Sp1/HDAC1-Mediated Transcriptional Regulation

Sp1 (specificity protein 1) is a ubiquitously expressed transcription factor of the mammalian Sp/KLF family that plays a crucial role in embryonic and early postnatal development [44]. E-cadherin is a master regulator of the epithelial phenotype, and an incessant downregulation of E-cadherin is required for the transition to mesenchymal morphology [15,45]. It is a transmembrane tumor suppressor protein that helps in cell-to-cell adherens junctions, and it is often localized adjacent to actin-containing filaments of the cytoskeleton. STRAP downregulates E-cadherin, disrupts paracrine signaling for cell growth, promotes tumorigenicity, and maintains mesenchymal morphology [46]. STRAP colocalizes with Sp1 in the nucleus and binds the C-terminal end of Sp1, resulting in the abrogation of the transcriptional activation of the E-cadherin gene. Silencing STRAP expression induces mesenchymal-to-epithelial transition (MET) via the upregulation of E-cadherin at the transcriptional level [46]. Moreover, STRAP deletion in mouse embryonic fibroblasts (MEFs) results in a loss of mesenchymal morphology and increased MET levels via the co-upregulation of Wilms tumor homolog 1 (WT1) and E-cadherin. Upon rescuing STRAP expression in these null cells, WT1 and E-cadherin expression are lost with consequent epithelial–mesenchymal transition [15].

Sp1 has also been shown to interact directly with epigenetic modulator HDAC1 (histone deacetylase (1), a chromatin remodelling factor, in order to regulate p21^Cip1^ expression [47]. p21^Cip1^, a cyclin-dependent kinase inhibitor 1 or CDK-interacting protein 1, inhibits the cyclin/CDK2 complex. The human p21^Cip1^ gene promoter contains two p53-binding and six Sp1-binding motifs. STRAP binds HDAC1 and recruits it to the Sp1-binding regions of the p21^Cip1^ promoter, leading to its transcriptional inhibition [46]. Furthermore, STRAP silencing results in the inhibition of Sp1 ubiquitination and, in turn, enhances stability, which has been directly correlated with increased expression of p21^Cip1^ and cell cycle arrest in the G1 phase of the cell cycle [46]. Collectively, STRAP downregulates E-cadherin and p21^Cip1^ expression through Sp1 inhibition and thereby increases tumorigenicity through EMT and the inhibition of cell growth arrest.

### 3.4. STRAP Promotes Colon Cancer Cell Stemness by Regulating MEK/ERK, Wnt/β-Catenin, and Notch Signaling

The MEK/ERK (Mitogen-activated ERK Kinase/Extracellular Signal-Regulated Kinase) pathway is activated upon extracellular growth factor (mitogen) ligand–receptor binding-mediated Ras/Raf activation, which leads to c-Myc-, c-Fos-, CREB-, MITF-, and C/EBP-induced transcriptional signaling for cell growth and proliferation [48]. Similarly, Wnt/β-Catenin signaling gets activated through Wnt ligand binding to the Frizzled/LRP5/6 membrane receptor complex. As a result, β-Catenin induces the transcription of genes like c-Myc, Cyclin D1, MMP-7, Axin-2, Survivin, CD44, etc., resulting in cell proliferation, invasion, and metastasis [49]. Adenomatous polyposis coli (APC) is a tumor suppressor protein and regulates β-Catenin signaling [50]. APC mutations are prevalent in colorectal cancer (CRC), resulting in the inhibition of β-Catenin degradation and induction of downstream oncogenic gene expression [14,51,52]. STRAP has been shown to activate Wnt/β-Catenin signaling to enhance tumorigenicity in APC-mutated CRC. More specifically, STRAP interacts with phospho-MEK1/2, recruits it from the cytosol to ERK1/2, and increases its phosphorylation by MEK1/2. Phospho-ERK1/2, in turn, phosphorylates and activates LRP6, a co-receptor of Wnt. Through this signaling, STRAP promotes the nuclear translocation of ß-Catenin and its target gene expression. Interestingly, STRAP is upregulated by mutated APC or activated Wnt/ß-Catenin signaling (Figure 1). Therefore, STRAP is a target of Apc mutation/deletion-induced intestinal tumorigenesis through a novel feedforward mechanism [14,51]. In addition, STRAP binds with GSK-3β and stabilizes β-Catenin by inhibiting its phosphorylation by GSK-3ß and ubiquitin-dependent degradation, resulting in stimulation of Wnt/β-Catenin signaling and the expression of its downstream targets in colorectal cancer cells [53].

In addition to β-Catenin, GSK-3β regulates the activation of various transcription factors/proteins involved in cell growth, proliferation, differentiation, polarity, and apoptosis, such as AP-1, c-Myc, c-Jun, nuclear factor kappa B (NF-κB), NFAT, Cyclin D1, Cyclin E, and Notch [54]. Among these, Notch proteins are involved in organogenesis and embryogenesis and induce cancer cell stemness properties [55]. STRAP interacts with Notch3 and stabilizes it by inhibiting its proteasomal degradation, and they are co-upregulated in non-small cell lung carcinoma [13]. Additionally, STRAP interacts with SUZ12 and modulates the polycomb repressive complex 2 (PRC2)-mediated methylation abundance on the loci of Notch target genes. STRAP overexpression increases levels of Notch target genes through transcriptional activation and maintains the cancer stem cell population (increased BMI1, CD24, CD44, EPCAM, CD29, and PSEN1), which may lead to enhanced chemoresistance. STRAP silencing showed the reduced expression of Notch receptors and its target genes, potentiated chemotherapeutic efficacy, and showed the inhibition of tumor growth in vitro and in vivo. These studies established a novel mechanism by which STRAP promotes colon cancer cell stemness and drug resistance by regulating Notch target genes [56]. Taken together, STRAP induces CRC growth, cell proliferation, and stemness by activating MEK/ERK, Notch, and Wnt/β-Catenin signaling.

### 3.5. STRAP Modulates the Function of the Ewing Sarcoma Protein

Ewing sarcoma (EWS) is a rare, aggressive, and metastatic cancer that develops in the soft tissues and bones and has the characteristics of a tiny round blue cell tumor [24,57]. EWS RNA-binding protein 1 (EWSR1) is a multifunctional protein that participates in a number of physiological functions, such as gene expression, cell signaling, RNA processing, and transport [58,59]. The protein has an RNA-binding domain at the C-terminus, a zinc finger domain, and an N-terminal transcriptional activation domain [59]. The chromosomal translocations between EWSR1 and various other transcription factors encoding genes result in the expression of chimeric proteins containing the N-terminal transcriptional activation domain of EWSR1 fused to the C-terminal DNA-binding domain of the transcription factor, which leads to Ewing Sarcoma [60]. These chimeric fusion proteins may function as both transcription activators and repressors. As an example, EWS-ETS represses the transcription of TβR-II, whereas EWS-FLI1 cooperates with CBP/p300 in order to induce transcription of Hepatocyte Nuclear Factor HNF4-dependent genes [24,61]. EWS activity has also been shown to be regulated by interaction with STRAP. EWS overexpression in cancer has been directly correlated with STRAP upregulation. Furthermore, STRAP has been shown to colocalize and interact with EWS in the nucleus through its N- and C-terminal domains. It disrupts the interaction between EWS and p300 and downregulates the EWS/p300-dependent activation of HNF4, ApoC3, and c-Fos [62]. Altogether, in addition to the MEK/ERK, β-Catenin, Notch, and TGF-β pathways, STRAP elevates the tumorigenic properties of EWS through its scaffold function.

### 3.6. STRAP Promotes the Malignant Phenotype in Neuroblastoma and Osteosarcoma

Neuroblastoma is a paediatric extracranial neuronal malignant cancer that causes approximately 15% of paediatric cancer-related deaths [63]. Increased STRAP expression has been shown to be associated with poor prognosis in neuroblastoma patients. STRAP silencing using small RNA (siRNA or shRNA) in neuroblastoma cells (SK-N-AS and SK-N-BE) resulted in decreased tumor cell viability, growth, stemness, and motility in vitro and tumor growth in vivo. Furthermore, knockout of STRAP in these cells has shown a decreased population in the S phase of the cell cycle, which reveals inhibited cell cycle progression. STRAP knockout downregulates various genes associated with tumor growth and progression, with notable downregulation of platelet-derived growth factor receptor ß (PDGFRβ), a highly upregulated oncogene in neuroblastoma. STRAP deletion correlates with reduced expression of stemness markers, including CD133, Oct4, Nanog, and Nestin [64], whereas its overexpression results in increased proliferation and stemness. Overexpression of STRAP increases the activation (through phosphorylation) of Focal Adhesion Kinase (FAK), a non-receptor protein tyrosine kinase, and promotes the growth and metastasis of neuroblastoma cells [65]. In addition to neuroblastoma, STRAP has also been found to be increased in various osteosarcoma cell lines (MNNG-HOS, 143B, and MG63), which is correlated to their oncogenic phenotype. STRAP silencing in these osteosarcoma cells resulted in inhibited invasion, migration, and re-implantation [66]. Collectively, STRAP levels are higher in some cancers, including neuroblastoma and osteosarcoma, which may contribute to the stimulation of tumor growth, invasion, and migration

### 3.7. STRAP in Hepatocellular Carcinoma and Intrahepatic Cholangiocarcinoma (IHCC)

Hepatocellular carcinoma (HCC), the sixth most common form of cancer in the world and one of the leading causes of cancer-related deaths, is caused by genetic and epigenetic changes that transform hepatocytes into malignant cells by disrupting signaling pathways [67,68]. STRAP has been implicated as an important oncogenic factor in hepatocellular carcinoma (HCC), specifically through its ability to modulate the Wnt/β-Catenin signaling pathway, which is crucial for cell proliferation, differentiation, and cancer progression. STRAP expression is significantly elevated in both human and mouse HCC tumor tissues compared to adjacent normal tissues, with its localization predominantly in the cytoplasm. CRISPR/Cas9 knockout studies in HCC cell lines Huh6 and Huh demonstrate that STRAP supports cell proliferation and tumor growth by amplifying β-Catenin signaling activity. Mechanistically, STRAP interacts with the GSK3β catalytic domain, leading to decreased β-Catenin phosphorylation and subsequent degradation, as previously described for colon cancer [53]. This results in the accumulation of active β-Catenin, which translocates to the nucleus and induces transcription of oncogenic target genes, including Cyclin D1 and c-Myc. STRAP-driven enhancement of β-Catenin signaling plays a crucial role in maintaining cancer stem cells, as demonstrated by the elevated expression of markers like LGR5 and AXIN2 in HCC cells. Additionally, the loss of STRAP in HCC cells leads to decreased metabolic activity and disruptions in cell cycle progression, underscoring its multifaceted role in driving hepatocellular carcinoma progression [69].

On the other hand, STRAP has emerged as an important oncogenic factor in intrahepatic cholangiocarcinoma. By interacting with circRNAs, such as circPCNXL2, it promotes tumor proliferation and metastasis. STRAP has been shown to form a complex with MEK1/2, which facilitates the activation of the ERK/MAPK signaling pathway, which is vital for the growth and survival of cells. According to a recent study, circPCNXL2 binds directly to STRAP, increasing the interaction between STRAP and MEK1/2, leading to increased ERK phosphorylation. The study of STRAP-associated pathways, especially their role in ERK/MAPK signaling in CRC and HCC, provides an avenue for therapeutic intervention, emphasizing their importance as both biomarkers and therapeutic targets for cholangiocarcinoma [70].

### 3.8. Role of STRAP in Non-Small Cell Lung Cancer (NSCLC) Progression

STRAP is markedly upregulated in non-small cell lung cancer (NSCLC) tissues and cell lines; however, functional knockdown of this protein significantly impairs cell migration, invasion, and tumor growth. Mechanistically, STRAP regulates the expression of XIAP, thereby suppressing apoptosis and promoting cell survival. The findings suggest that STRAP may be a potential oncogenic driver of NSCLC and a potential therapeutic target [71].

### 3.9. STRAP Is a Strong Predictor of an Unfavorable Effect of 5-FU-Based Adjuvant Chemotherapy in Colorectal Cancer

Molecular markers predicting the benefit of adjuvant chemotherapy in cancer patients are urgently needed. To evaluate the prognostic and predictive significance of STRAP, it was observed that the STRAP gene is amplified in 22.8% of colorectal tumors. In patients who did not receive adjuvant chemotherapy, STRAP amplification was associated with improved outcomes. These patients had worse survival when treated with adjuvant therapy in comparison with patients without chemotherapy. In contrast, patients with diploidy or the deletion of STRAP benefited from chemotherapy. These findings suggest that STRAP amplification may serve as a negative prognostic marker for 5-FU-based adjuvant chemotherapy, and its copy status might be a useful parameter in the management of patients with Stage II and Stage III colorectal cancers [72].

## 4. STRAP Regulates Non-Oncogenic Functions Such as mRNA Splicing, CAP-Independent Translation, and Embryonic Development

### 4.1. STRAP Stabilizes NF-κB and Activates Its Signaling by Interacting with TAK1/IKK

Most cancer cells have high basal activity of nuclear NF-κB that constantly drives their proliferation. NF-κB exists predominantly as a p65/p50 heterodimer and remains sequestered with an inhibitory subunit of NF-κB (IκBα) in the cytoplasm. Ligands such as TNFα and IFNγ induce the activation of the IκBα kinase (IKK) complex by phosphorylation. Activated IKK phosphorylates IκBα, leading to its ubiquitination and proteasomal degradation. Thus, the released active p65/p50 heterodimer translocates to the nucleus and regulates target gene expression, most of which participate in cell cycle progression [73]. STRAP has been reported to regulate the TLR2/4 signaling pathway via the formation of a scaffold with TAK1, IKKα, and p65. STRAP binds TAK1, IKKα, and p65 in a complex in which TAK1 phosphorylates IKKα and consequently activates p65. Furthermore, STRAP regulates TLR2/4-induced IL-1β, IL-6, and TNFα expression by facilitating NF-κB phosphorylation and subsequent nuclear translocation [74]. STRAP also regulates TLR3-mediated IFNγ expression by interacting with IFN regulatory factor 3 (IRF3) in the TAK1/IKKα/p65 complex and induces target gene expression [75]. Since, STRAP expression is found to be high in most of the cancers, and NF-κB signaling is also directly linked to aggressive cancers; thus, STRAP-mediated NF-κB regulation may contribute to its oncogenic activity.

### 4.2. STRAP Activates NM23-H1 Through Scaffold Formation and Inhibits ASK1-Induced Apoptosis

**Figure 2:**
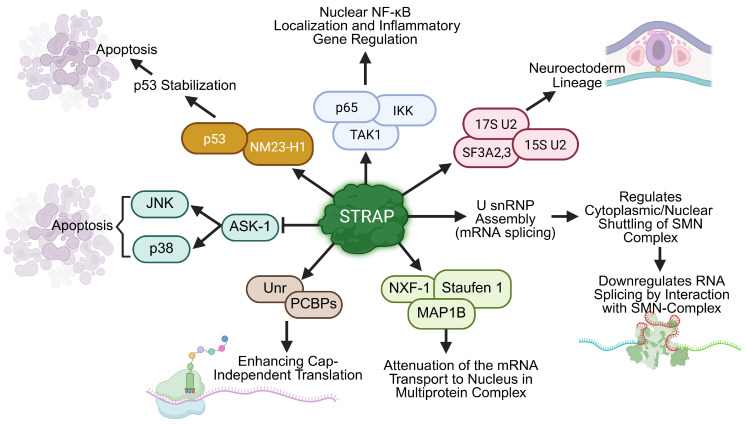
The role of STRAP in apoptosis, inflammation, and RNA regulation highlights its importance in cellular homeostasis and development. STRAP promotes apoptosis by stabilizing p53 through its interaction with NM23-H1, enhancing apoptotic signaling. STRAP inhibits ASK1 activation, which functions as an upstream activator of the stress kinases, leading to an induction in apoptosis. It modulates inflammatory signaling by interacting with NF-κB components, including p65, TAK1, and IKK. It further enhances cap-independent translation by interacting with Unr and PCBP proteins. STRAP plays a critical role in regulating alternative splicing (AS) during neural differentiation. It interacts with SF3A2,3, 15S U2, and 17S U2 to facilitate exon selection and spliceosome assembly, and its deletion disrupts lineage commitment in embryoid bodies (EBs), which indicates its pivotal role in neuroectoderm differentiation. At the post-transcriptional level, STRAP regulates U snRNP assembly by interacting with SMN complex components (Gemin 6, Gemin 7, SmB, SmD2, and SmD3) and downregulating RNA splicing. Predominantly localized in the cytoplasm, STRAP facilitates the cytoplasmic-nuclear shuttling of the SMN complex, which is essential for pre-mRNA splicing and spliceosome assembly. STRAP attenuates mRNA transport to the nucleus in multiprotein complexes through its interaction with NXF-1, Staufen1, and MAP1B. These interactions ensure the fidelity of mRNA processing and transport. In summary, this figure illustrates the multifaceted roles of STRAP in regulating developmental, apoptotic, and inflammatory processes.

NM23-H1 is involved in nucleotide metabolism, specifically pyrimidine metabolism. Although it is popularly known as a metastasis suppressor, it has also been reported to be involved in proliferation, differentiation, apoptosis, development, and endocytosis [76]. NM23-H1 has been reported to interact with STRAP, and this interaction potentiates NM23-H1-mediated cell growth and STRAP-mediated TGF-β signaling inhibition [77]. Furthermore, STRAP interacts with p53 alone and in a complex with NM23-H1 and enhances p53 stabilization by inhibiting MDM2-mediated p53 suppression. Under stress conditions, the STRAP/NM23-H1/p53 ternary complex increases the nuclear localization of p53 and upregulates the expression of effector genes [78]. As a tumor suppressor, ASK1 induces apoptosis in breast and lung cancer cell lines. The association between STRAP and ASK1 inhibits apoptosis by reducing its kinase activity (Table 1). Therefore, the oncogenic functions of STRAP are regulated through interactions with many cellular factors/proteins and by upstream signaling, depending on the in vitro experimental context (Figure 2).

### 4.3. STRAP Is Involved in mRNA Splicing, Nuclear Transport, and CAP-Independent mRNA Translation

The macromolecular survival motor neuron complex (SMN complex) mediates the active process of spliceosomal small ribonucleoprotein (U-rich snRNP) assembly for pre-mRNA splicing. The SMN complex contains the SMN protein and six additional proteins named Gemins 2–7 [79,80]. A mutation in the SMN protein is involved in the development of spinal muscular atrophy in patients. STRAP downregulates the snRNP complex assembly by interacting with Gemin 6, Gemin 7, SmB, SmD2, and SmD3, components of the SMN complex [79,80]. As STRAP is predominantly found in the cytoplasm, it may play an important role in the movement of the SMN complex from the nucleus to the cytoplasm (Figure 2).

In eukaryotes, most nuclear export of mRNA is facilitated by evolutionarily conserved nuclear export factor (NXF) receptors. The mouse analogue of NXF1, mNXF7, interacts with brain-specific microtubule-associated protein 1B (MAP1B) and Staufen1 (Stau1) in association with STRAP to form a multiprotein complex for nuclear export of mRNA in neuronal cells [81]. These studies reveal a network of interactions of several proteins that culminates in nuclear export and cytoplasmic trafficking of mRNAs.

Unr (upstream of N-ras) is a cytoplasmic RNA-binding protein that contains five cold shock domains (CSD) and has been implicated in cap-independent translation of multiple proteins. The unr protein plays a crucial role in initiating translation driven by the HRV-IRES element in picornavirus RNAs [82]. STRAP, also known as unrip (Unr-interacting protein), together with Unr and other poly (rC)-binding proteins (PCBPs), increases c-Myc internal ribosomal entry site (IRES) translation stimulation activity [83]. Overall, STRAP interferes with protein expression through (a) reducing RNA splicing by interacting with the SMN complex, (b) facilitating mRNA transport to the cytoplasm, and (c) enhancing cap-independent translation by binding Unr.

### 4.4. STRAP Regulates the Splicing Networks of Lineage Commitment That Are Involved in Embryogenesis

In an attempt to determine the cause of embryonic lethality in Strap-null mice and its functional role in developmental stem cells, we identified STRAP as a putative spliceosome-associated factor and thoroughly investigated its role in alternative splicing (AS) events during early development. A study using enhanced-CLIP sequencing (eCLIP-seq) has demonstrated that STRAP binds to specific exonic and intronic regions of genes involved in the development of the nervous system in mouse embryonic stem cells (mESCs) and embryos [16]. Interestingly, this binding is highly position-specific, occurring predominantly near 5′ and 3′ splice sites, influencing exon inclusion and skipping, as demonstrated for two neuronal-specific genes, *Nnat* and *Mark3*. STRAP induces the skipping of exon 2 in Nnat, which produces isoforms necessary for neural patterning. Similarly, STRAP regulates Mark3 splicing by balancing isoforms associated with neuronal progenitors and differentiated neural cells. The deletion of Strap results in the appearance of numerous cassette exons (Cas) in mouse embryoid bodies (EBs) that are in the process of undergoing a neuroectoderm-like transformation (Figure 2). Further, the loss of STRAP leads to a significant disruption of AS patterns, a halt in lineage differentiation, a delay in the closure of the neural tube, and altered exon skipping in Xenopus. STRAP also interacts with U2 snRNP proteins and facilitates the assembly of the 17S U2 snRNP complex, which ensures splicing accuracy [16]. Collectively, these findings reveal a previously unknown function of STRAP in mediating the splicing networks of lineage commitment, the alteration of which is involved in embryonic lethality of *Strap*-null mice.

### 4.5. Emerging Role of STRAP in Cellular Stress and Immune Responses

A recent study identified a novel role for STRAP in DNA damage response by identifying it as a binding partner of N1-methyldeoxyadenosine (1mdA) and demonstrating that it helps to promote the repair of this alkylation-induced DNA lesion. STRAP knockdown decreased 1mdA repair and played a role in transcriptional bypass efficiency, indicating that it is necessary for the maintenance of the genome and transcription under genotoxic stress conditions [84].

Additionally, STRAP has been identified as a positive regulator of the innate immune response that appears to be triggered by pseudorabies virus (PRV) by modulating type I interferon (IFN-I) signaling. STRAP interacts with TBK1, stabilizing it and enhancing IRF3 phosphorylation. STRAP promotes IFN-I-mediated antiviral gene expression by preventing TBK1 degradation mediated by the viral protein UL50, revealing a novel role in host antiviral defense [85].

## 5. Discussion

Most of the WD40 domain-containing proteins form a scaffold with key proteins to activate different signaling pathways that are required for divergent cellular functions. One such protein, STRAP, is found to be upregulated in most epithelial cancers and has been directly correlated with poor prognosis in cancer patients. In colorectal cancer patients, the upregulation of STRAP is associated with worse survival following adjuvant therapy. In contrast, patients carrying tumors with normal or low STRAP expression benefited from the treatment, suggesting its direct involvement in colorectal cancer progression and chemoresistance [56,72]. The above discussions of in vitro experiments suggest that STRAP regulates various oncogenic signaling pathways, leading to cell growth, migration, invasion, EMT, etc. In support of these observations, in vivo xenograft, allograft, and genetic mouse models have shown that the upregulation of STRAP expression promotes tumor initiation, progression, and metastasis [14,56]. Although STRAP is crucial for embryonic stem cell differentiation and development through alternate splicing [15], its upregulation maintains intestinal cancer cell stemness, leading to tumorigenic functions [14]. It remains to be determined how different oncogenic signaling pathways regulated by STRAP are integrated together in different stages of cancer progression, depending on the genetic mutations.

Although STRAP was originally cloned using the cytoplasmic domain of TßR-I as a bait in a yeast two-hybrid screen [20] and is largely known as an inhibitor of classical TGF-ß/Smad signaling, it is now apparent that STRAP’s biological functions are not limited to TGF-beta signaling. STRAP interacts with inhibitory Smad, Smad7, and forms a scaffold with TßR-I and TßR-II, which results in the inhibition of Smad2/3 phosphorylation and subsequent TGF-ß/Smad signaling. In addition, STRAP/PDK-1 interaction upregulates PDK-1 activity and further enhances TβR-I/TßR-II/Smad7 interactions and potentiates Smad pathway inhibition. TGF-β induces apoptosis and inhibits cell proliferation in the early stage of cancer progression through Smad signaling. Several lines of evidence suggest that carcinoma cells frequently lose the tumor suppressor functions of TGF-ß. The upregulation of TGF-ß signaling inhibitors like STRAP and Smad7 and their synergistic function present a novel intracellular mechanism by which a portion of human tumors become refractory to the antitumor effects of TGF-ß [2]. Additionally, TGF-β/Smad signaling also regulates cyclin-dependent kinase inhibitors (CDKIs), including members of the Ink4 family (p^15^, p^16^) and Cip/Kip family (p21^Cip1^, p27^KIP1^), and these CDKIs are involved in various pathways to inhibit cell proliferation. STRAP, through TGFβ/Smad inhibition, tunes cellular CDKI levels and induces cell proliferation. It has been shown that in advanced cancers, TGF-ß/Smad-mediated growth suppression functions are abolished. At that time, TGF-ß induces EMT and promotes tumor growth and metastasis through the activation of MEK/ERK, P38 MAPK, JNK, and PI3K pathways. However, STRAP does not inhibit these growth-promoting TGF-ß functions in the late stage. Comprehensive future studies will be required to decipher these critical questions.

APC deletions/mutations are involved in the aberrant activation of Wnt/β-Catenin signaling in about 80% of sporadic colon cancers. APC inactivation leads to β-Catenin stabilization and, consequently, to deregulation of the Wnt pathway through interaction with TCF/LEF targets. Importantly, the crosstalk between Wnt/β-Catenin signaling and other pathways complicates the possibility of targeting Wnt/β-Catenin signaling in patients with APC mutations. Apart from the TGF-β/Smad pathway, STRAP-induced ERK phosphorylation leads to effector gene expression and LRP6-mediated activation of Wnt/β-Catenin signaling. STRAP has been shown to be upregulated in more than 50% of colon adenomas, and the inactivation of APC or the activation of Wnt/ß-Catenin signaling increases the expression of STRAP. These two signaling pathways maintain the stemness phenotype induced by APC mutation through a feed-forward mechanism, STRAP/MEK-ERK/Wnt-β-Catenin/STRAP, in which STRAP is a critical factor [14]. Therefore, future studies will determine whether STRAP is a molecular target for the treatment of colon cancer in patients with APC mutation or Wnt/β-Catenin activation. A recent study suggests that cancer should also be considered as an ecological disease, in which tumor progression is attributable to the dynamic interaction between cancer cells and their surrounding microenvironment [86]. They proposed a model of “unity of ecology and evolution,” in which cancer is viewed as an evolving spatiotemporal pathological ecosystem. In this ecological pathology, tumor behavior is determined by a variety of factors, including genetic mutations, intercellular competition, environmental stress, immune evasion, and niche formation. STRAP, through its role in signaling pathways and interaction with key regulators of proliferation, EMT, and stemness, may play a critical role in modulating this ecosystem. Incorporating an ecological framework may help explain phenomena such as tumor plasticity, therapeutic resistance, and metastatic dispersal, areas that remain only partially understood at the molecular level. The combination of STRAP protein biology with tumor ecology may allow for the discovery of novel therapeutic targets that can disrupt the cancer ecosystem [86].

## 6. Conclusions

STRAP is a potent oncogene, initially identified as an inhibitor of TGF-ß signaling, and plays a critical role in the activation of several tumorigenic and metastatic pathways, including Notch, MEK/ERK, PKB/AKT, ASK1, and Wnt/ß-Catenin signaling, contributing to cancer cell stemness and therapeutic resistance. Apart from its anti-apoptotic and pro-survival functions, STRAP has been shown to mediate cell death in cell culture studies depending on the stimuli, stress conditions, changes in the redox status, and on the mutational status of p53 [78,87]. However, these few studies were mostly performed on cultured cells. The tumor-suppressive role of STRAP during tumorigenesis (if any) should be tested using in vivo genetic experiments and human tumor specimens. With these contrasting reports on the roles of STRAP in cancer, the tumor-promoting effects of STRAP, including the induction of cell proliferation, survival, migration, invasion, cancer cell stemness, metastasis, and the inhibition of apoptosis, were established using in vivo models and verified with human tumor tissues. Therefore, the upregulation of STRAP in solid tumors, coupled with its various pro-oncogenic functions and crosstalk with oncogenic signaling, provides a strong rationale for it to be a potentially important drug target for therapeutic intervention in human cancers.

## Figures and Tables

**Table 1:** STRAP-associated proteins and complexes involved in physiological and oncogenic processes.

STRAP Interacting Proteins/Complex	Description	STRAP-Mediated Functional Changes in Relevance to Carcinogenesis	Refs.
TGFß-RI/TGFß-RII	Transmembrane serine/threonine receptor kinases	Formation of a ternary complex that inhibits TGF-ß signaling	[1,2,24]
Smad7	Smad2/3/4 inhibitor (SMAD family member 7)	Inhibits nuclear localization of Smad2/3/4 complex and TGF-ß/Smad signaling	[2,25]
MEK1/2	Dual-specificity protein kinases	Increases MEK/ERK and Wnt/ß-Catenin signaling	[14,35]
GSK-3ß	Serine/threonine protein kinase	Stabilization of ß-Catenin and stimulation of Wnt/ß-Catenin signaling in CRC cells	[37]
Notch3	A transmembrane protein that acts as a cell surface receptor	Stabilizes Notch3 by inhibiting its proteasomal degradation and promotes lung tumorigenesis	[13]
SUZ12	A component of the polycomb repressor complex 2 (PRC2)	Regulates Notch signaling gene expression by modulating methylation	[40]
Sp1	A zinc finger transcription factor in the Sp/KLF family	Abrogation of the transcriptional activation of E-cadherin and p21^Cip1^ and subsequent regulation of EMT and cell proliferation, respectively	[43]
HDAC1	A histone deacetylase that regulates physiological processes	Regulates its binding to the Sp1-binding region (C-terminal domain) of p21^Cip1^ promoter, downregulating its expression	[43,44]
PDK-1	A phospho-inositide-dependent kinase	Positively regulates PDK1 activity, inhibition of TNF-α/TGF-ß mediated cell apoptosis	[55]
ASK-1	A member of MAP kinase family	Phosphorylation of ASK-1(Thr/Ser) leads to inhibition of ASK1-mediated cell death	[55,56]
EWS	The Ewing Sarcoma (EWS) is a multifaceted oncogenic RNA-binding protein (RBP)	Attenuates EWS/p300-dependent activation of HNF4, ApoC3, and c-Fos in the nucleus and elevates the oncogenic properties of EWS	[62]
TAK1/IKK-α/p65 complex	P65 is a component of the NF-κB transcription factor complex TAK1/IKK-α is a kinase complex in the core element of the NF-κB cascade	Formation of scaffold with TAK1, IKKα, and p65, facilitates NF-κB nuclear translocation and inflammatory genes regulation	[68]
Gemin 6/7, SmB, SmD2 and SmD3 components of SMN complex	Nuclear ribonucleoproteins in the RNA–protein complex that participate in the splicing of pre-mRNAs	Downregulates snRNP complex assembly and regulates pre-mRNA splicing	[78,79]
NXF1/MAP1B/Stau1 Complex	Nuclear RNA export factor 1, Microtubule-associated protein 1B, and Staufen1 are proteins that interact with each other to facilitate mRNA transport	Forms a multiprotein complex and facilitates nuclear mRNA export in neuronal cells	[80]
Unr/PCBPs complex	Members of the cytoplasmic RNA-binding protein family	Increases c-Myc internal ribosomal entry site (IRES) translation and enhances cap-independent translation	[81,82]
NM23-H1/P53	p53 is the guardian of the genome and a tumor suppressor protein. NM23-H1 is a metastasis suppressor	Enhances p53 stabilization and upregulates effector gene expression, may promote apoptosis and cell cycle regulation	[72]
B-MYB	G1/S phase transcription factor of the MYB family	Inhibition of nuclear localization of Smad3, increase in p53 nuclear localization, and suppression of TGF-ß mediated growth inhibition and apoptosis	[83]

## Data Availability

No new data were generated or analyzed in the course of this study.
